# Ca^2+^-Dependent and Ca^2+^-Independent ATP Release in Astrocytes

**DOI:** 10.3389/fnmol.2018.00224

**Published:** 2018-07-02

**Authors:** Yingfei Xiong, Suhua Sun, Sasa Teng, Mu Jin, Zhuan Zhou

**Affiliations:** ^1^State Key Laboratory of Biomembrane and Membrane Biotechnology and Peking-Tsinghua Center for Life Sciences and PKU-IDG/McGovern Institute for Brain Research, Institute of Molecular Medicine, Peking University, Beijing, China; ^2^Department of Neurosurgery, Affiliated Hospital of The Air Force Institute of Aeromedicine, Beijing, China; ^3^Department of Anesthesiology, Beijing Friendship Hospital, Capital Medical University, Beijing, China

**Keywords:** glial transmitter, ATP, astrocyte, exocytosis, P2X7, calcium, mechanical stimulation

Like neurons, astrocytes are abundant in the central nervous system. They contact all types of cells in the brain, communicate with them, and modulate their activity by releasing gliotransmitters, including glutamate, ATP, etc. (Newman, 2003; Halassa and Haydon, 2010; Hamilton and Attwell, 2010; Volterra et al., 2014; Verkhratsky and Nedergaard, 2018), although the role of astrocytes in neurotransmission is still debated (Nedergaard and Verkhratsky, 2012; Fiacco and McCarthy, 2018).

ATP is considered to be a powerful extracellular messenger in both the peripheral and central nervous systems (Edwards et al., 1992; Fields and Stevens, 2000; Burnstock, 2007; Verkhratsky et al., 2009). *Via* activating multiple receptors in glia and neurons, ATP signaling participates in many important functions including cell development and synaptic plasticity (Evans et al., 1992; Newman, 2003; Zhang et al., 2003; Agresti et al., 2005; Abbracchio et al., 2009; Butt, 2011; Wurm et al., 2011; Verkhratsky et al., 2016). However, unlike the mechanism of neurotransmission *via* quantal exocytosis (Katz, 1959, 1969; Augustine and Neher, 1992; Neher, 1998; Sudhof, 2004; Pankratov et al., 2006, 2007; Sudhof and Rothman, 2009), the mechanisms by which ATP is released remain controversial. Because ATP is easily hydrolyzed, monitoring its real-time release is a challenge.

## Early studies of non-quantal ATP release from astrocytes

**Figure 1:**
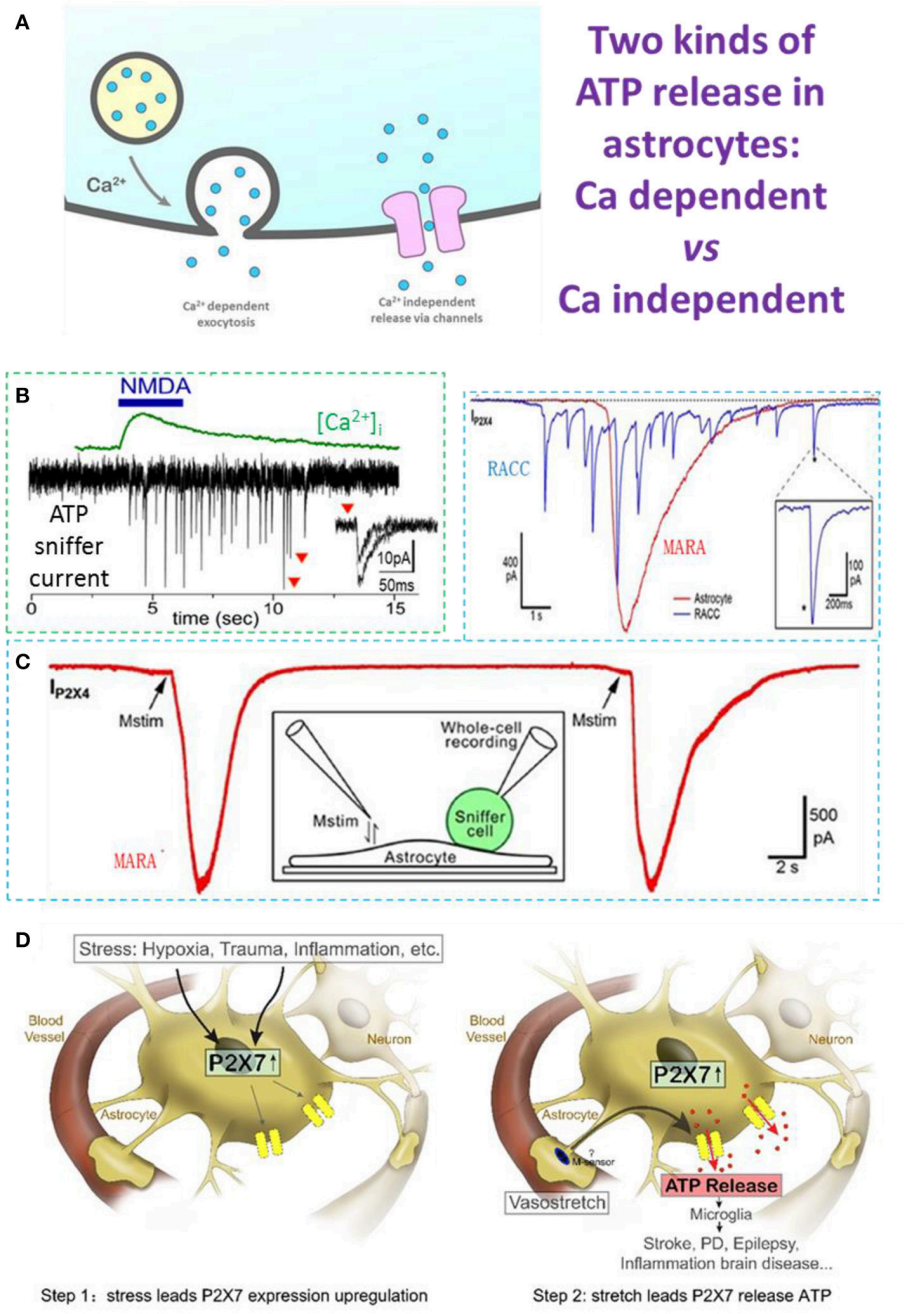
Ca^2+^-dependent and Ca^2+^-independent ATP release in astrocytes. **(A)** Two types of ATP release: Ca^2+^-dependent quantal release and Ca^2+^-independent non-quantal release. In the Ca^2+^-dependent pathway, secretory vesicles with packaged ATP are trafficked to the plasma membrane where they dock and fuse with it on arrival of a stimulus; this typically depends on an increase of cytosolic Ca^2+^. In the Ca^2+^-independent pathway, ATP is released through channels expressed on the astrocyte plasma membrane, such as the swelling-induced anion channel, connexin hemichannels activated by lower Ca^2+^ concentrations, and ionotropic purinergic receptor channels. **(B)** N-methyl-D-aspartate (NMDA)-induced Ca^2+^-dependent quantal ATP release from a freshly-isolated astrocyte, recorded by a HEK293-P2X2 sniffer cell. **(C)** Typical MARA current (IP2X4) recorded with an HEK293A sniffer-cell expressing P2X4 (ATP sniffer) on an astrocyte. The astrocyte was stimulated twice and the MARA signal was reproducible. Inset, cartoon of the experimental protocol for MARA recording; Upper right: MARA vs Ca^2+^-dependent ATP release of burst quantal events from a rat chromaffin cell (RACC). **(D)** Two-step hypothesis of MARA-mediated brain diseases. Left. Step 1: Stress induces upregulation of P2X7 receptor expression in astrocytes. The stressors include hypoxia (i.e., stroke and ischemia), trauma, and CNS diseases associated with inflammation or neurodegeneration. Right. Step 2: Stretch leads to P2X7-mediated ATP release from astrocytes. The abundant ATP is an emergency signal for the neural immune system that recruits and activates microglia in the ATP “hot-spots” and promotes disease. All data adapted from Xiong et al. (2018), except B adapted from Lalo et al. (2014). All data with reproduction permission from the original publishers.

Early studies usually applied indirect methods such as dye-uptake, ATP analog labeling, and luciferase-luciferin system tests to indirectly detect ATP release (Duan et al., 2003). Based on these studies, ATP was thought to be released through specific channels, such as connexin/pannexin hemichannels, “maxi-anion” channels, and P2X7 receptor channels (Figure 1A, Stout et al., 2002; Duan et al., 2003; Bao et al., 2004; Suadicani et al., 2006; Kang et al., 2008; Liu et al., 2008; Iglesias et al., 2009; Bennett et al., 2012). These studies used indirect measurements based on measuring dyes or currents through channels that can pass molecules larger than ATP.

## Quantal ATP release under physiological conditions

Patch-clamp recording *via* sniffer cells (Young and Poo, 1983; Liu et al., 2011) is a powerful tool with which to investigate the ATP release mechanism due to its high spatiotemporal resolution (Hollins and Ikeda, 1997; Pangrsic et al., 2007; Karanauskaite et al., 2009; Lalo et al., 2014; Liu et al., 2014; Lee et al., 2015). By using ATP-sniffer cells, Lalo et al. recorded quantal ATP release in astrocytes freshly isolated from mouse cortex. ATP is released by Ca^2+^-dependent exocytosis following the activation of metabotropic and ionotropic receptors or direct UV-uncaging. The ATP release is SNARE protein-dependent and is eliminated by pretreatment with bafilomycin, a blocker of vacuolar-type H-ATPase. The kinetics of sniffer-cell responses are consistent with the millisecond time-scale, suggesting that ATP exocytosis is from synaptic-like small vesicles (Figures 1A,B). The ATP released from astrocytes (1) activates P2X receptors in neighboring neurons to enhance excitatory signaling, and (2) down-regulates inhibitory synaptic signaling (Lalo et al., 2014).

In addition to the tiny quantal ATP release arising from Ca^2+^-dependent exocytosis in freshly-isolated astrocytes, an earlier report proposed that Ca^2+^-dependent lysosome exocytosis is responsible for quantal ATP release in cultured astrocytes (Zhang et al., 2007). They provided three lines of indirect evidence: (1) the uptake of a fluorescent ATP analog, MANT-ATP, into lysosomes; (2) the presence of ATP in biochemically-purified lysosomes; and (3) the real-time visualization of Ca^2+^-dependent lysosome exocytosis by total internal reflection fluorescence microscopy imaging of a false neurotransmitter (FM2-10). Although these data raised the possibility of quantal ATP release *via* lysosomal exocytosis, direct recording of quantal ATP release from glial lysosomes was absent.

## Stretch-induced Ca^2+^-independent ATP release through P2X7 channels

It was our original goal to record quantal ATP release in cultured hippocampal astrocytes using ATP-sniffer cells. To our surprise, with three independent assays of lysosomal exocytosis, our real-time sniffer recordings clearly denied ATP release by Ca^2+^-dependent lysosomal exocytosis (Xiong et al., 2018). Instead, following a gentle membrane stretch, Ca^2+^-independent non-vesicular ATP release occurred in the cultured astrocytes (Xiong et al., 2018). In contrast to the quantal spikes of ATP release in chromaffin cells, the Mechanically-induced ATP Release from Astrocytes (MARA) displays a single spike with distinct kinetic characteristics. MARA is ~300-fold greater in release content and ~50 times longer in duration (Figure 1C, upper right inset). Mechanistically, MARA-mediated ATP release is (1) Ca^2+^-independent, (2) not *via* lysosome exocytosis, and (3) mitochondria-dependent (Xiong et al., 2018). The P2X7 receptor channel is essential for MARA-mediated ATP release, because it is profoundly inhibited by Brilliant Blue G, a selective P2X7 antagonist, as well as by RNA interference-based P2X7 knockdown (Xiong et al., 2018). Considering that the open pore of the P2X7 channel allows the permeation of cytosolic molecules of molecular weight ≤ 900 Da, P2X7 channels should be able to release ATP (507 Da) (Yan et al., 2008; Nagasawa et al., 2009), and other purines, such as AMP, ADP, or adenosine. Together, MARA occurs when mechanical stretch triggers ATP efflux through P2X7 channel pores in “activated” astrocytes expressing P2X7 receptors in culture/*in vitro* or hypoxia/trauma/disease *in vivo* (Nagasawa et al., 2009). Hypoxia might decrease mitochondrial-dependent cytosolic ATP level and partially compensate the MARA signal for the increase in P2X7 expression.

One important open question left by Xiong (Xiong et al., 2018) is the identity of the mechanical sensor by which MARA initiates mechanical-P2X7-ATP release in astrocytes. Since P2X7 itself is not mechanosensor, we hypothesize that a mechanosensor [such as piezo 1 protein (Zhao et al., 2018)] binds P2X7 and “transactivates” mechanical force to activate P2X7 [One recent example of protein-transactivation is that a voltage-sensor channel activates another binding protein of vesicle fusion-pore (Chai et al., 2017)]. Following the discovery of MARA, identification of this sensor is critical for treating possible MARA-mediated brain diseases as proposed below. In addition to P2X7 (Xiong et al., 2018), hemichannels such as connexins (Stout et al., 2002) and large conductance Cl^−^ channel (Liu et al., 2008) have been reported to mediate the non-vesicular ATP release from astrocytes. At present it is unclear about the relative contributions to ATP release among these channel types, because the stimulations in these studies were different. These ATP-release pathways through different channels may play condition-dependent roles in astrocytes' functions.

## A hypothesis of MARA in brain protection and diseases

In contrast to the Ca^2+^-dependent quantal ATP release in freshly-isolated astrocytes, the Ca^2+^-independent ATP release event (MARA) in cultured astrocytes is ~10,000 times greater (Figure 1C). P2X7 is up-regulated under ischemic conditions *in vivo* (Nagasawa et al., 2009), and this could contribute to the release of large amounts of ATP from cellular sources, and in the extracellular space it is quickly hydrolyzed to ADP, AMP, and adenosine, which activate their receptors and play roles in brain protection and damage (Neary et al., 2003; Choo et al., 2013; Rodrigues et al., 2015). On the other hand, the large ATP release *via* MARA would recruit microglia, leading to protective or pathological pathways (Dou et al., 2012). Thus, we propose that MARA could be a mechanism underlying brain diseases such as those associated with hypoxia/ischemia and trauma, as well as other neurological disorders (Parkinson's disease, Alzheimer's disease, and epilepsy) (Figure 1D). Our hypothesis is detailed as follows. The first step is MARA genesis, which is dependent on P2X7 receptor expression. The expression level of P2X7 receptors in astrocytes is up-regulated under either of the two conditions: (1) in culture, which is an extremely stressful condition lacking blood and other support for astrocytes (Narcisse et al., 2005; Bartlett et al., 2014; Burnstock, 2017; Xiong et al., 2018); and (2) *in vivo*, when astrocytes suffer stresses, such as hypoxia (i.e., ischemia and stroke), trauma, or some CNS diseases (for example, inflammation and/or neurodegeneration; Ballerini et al., 1996; Narcisse et al., 2005; Nagasawa et al., 2009; Burnstock, 2017). Indeed, few astrocytes express P2X7 receptors in the intact hippocampal slices under physiological conditions (Nagasawa et al., 2009; Xiong et al., 2018). As the P2X7 receptor is a key modulator of aerobic glycolysis, it has an intrinsic ability to reprogram cell metabolism to meet the needs imposed by adverse environmental conditions (Amoroso et al., 2012). The up-regulation of P2X7 receptors in astrocytes is not only a form of adaptation to stress, but is also a necessary preparation for MARA to execute its repair function in its brain region (Figure 1D, step 1). The second step is MARA activation following mechanical stimulation, which could be generated by arterioles *in vivo*. Astrocytes regulate cerebral blood flow to match the metabolic requirements of the brain (Gordon et al., 2008) by eliciting the vasoconstriction or vasodilation of arterioles. A tension change due to such vasoconstriction/dilation is an effective physiological mechanical stimulus (membrane stretch) for astrocytes, which are activated by deformation of their surroundings on a timescale of milliseconds (Janmey and Miller, 2011). Physiological arteriole stretch does not trigger MARA because P2X7 receptors are rare in astrocytes under normal conditions. When stresses such as hypoxia/trauma occur, the expression of astrocytic P2X7 receptors is up-regulated, and if the stress persists and exceeds a threshold, the abnormal changes in arteriole tension directly trigger MARA (Figure 1D, step 2). So, noxious stimuli up-regulate the P2X7 receptor expression in astrocytes, and this can be considered as an adaptive change in response to stress and the beginning of pathological damage, because up-regulation of P2X7 receptor expression is the prerequisite for non-quantal ATP release/MARA. When the steady-state is disturbed, MARA occurs and triggers a sustained increase in extracellular ATP, which then acts to alert the presence of a “hot spot.” This signal recruits and activates microglia, which scavenge and phagocytize injured cells and cellular debris, increase the susceptibility of neurons to damage, promote astrogliosis, and mount neuroinflammatory responses (Parpura et al., 2012; Rivera et al., 2016). In this sense, both astrocytes and microglia comprise the immune system of the CNS: each of a vast number astrocytes performs an immobile surveillance for its specific location in the brain, signaling stress, while each microglia cell acts as a mobile patrol, responding to the astrocytic “alert” signal—an unusually large increase in extracellular ATP concentration. To a certain extent, MARA from a few astrocytes recruits a few microglia to clear the site and protect the brain. However, when the stress is excessive, MARA from a large number of astrocytes may recruit too many microglia, overkill healthy tissue, and cause irreversible brain damage (disease).

## Author contributions

All authors listed have made a substantial, direct and intellectual contribution to the work, and approved it for publication.

### Conflict of interest statement

The authors declare that the research was conducted in the absence of any commercial or financial relationships that could be construed as a potential conflict of interest.
